# A simulation study for geographic cluster detection analysis on population-based health survey data using spatial scan statistics

**DOI:** 10.1186/s12942-022-00311-6

**Published:** 2022-09-09

**Authors:** Jisu Moon, Inkyung Jung

**Affiliations:** Division of Biostatistics, Department of Biomedical Systems Informatics, Yonsei University College of Medicine, 50-1 Yonsei-ro, Seodaemun-gu, Seoul, 03722 Korea

**Keywords:** Health survey, Geographic surveillance, Sampling design, Sampling weight, Spatial cluster detection

## Abstract

**Background:**

In public health and epidemiology, spatial scan statistics can be used to identify spatial cluster patterns of health-related outcomes from population-based health survey data. Although it is appropriate to consider the complex sample design and sampling weight when analyzing complex sample survey data, the observed survey responses without these considerations are often used in many studies related to spatial cluster detection.

**Methods:**

We conducted a simulation study to investigate which data type from complex survey data is more suitable for use by comparing the spatial cluster detection results of three approaches: (1) individual-level data, (2) weighted individual-level data, and (3) aggregated data.

**Results:**

The results of the spatial cluster detection varied depending on the data type. To compare the performance of spatial cluster detection, sensitivity and positive predictive value (PPV) were evaluated over 100 iterations. The average sensitivity was high for all three approaches, but the average PPV was higher when using aggregated data than when using individual-level data with or without sampling weights.

**Conclusions:**

Through the simulation study, we found that use of aggregate-level data is more appropriate than other types of data, when searching for spatial clusters using spatial scan statistics on population-based health survey data.

## Introduction

It is important to identify geographical disparities in health outcomes related to chronic diseases [1], physical activity [2], behavioral health [3], and mental health [4]. In particular, identifying locations with significantly high- or low-risk health outcomes would be useful for guiding targeted health programs and shaping health policies to reduce health disparities [5]. Health authorities often conduct health surveys of the general population; thus, it might help analyze the spatial cluster patterns using this data.

Among the various statistical methods for geographic cluster detection, the spatial scan statistic proposed by Kulldorff [6] has been widely used in various epidemiologic studies. This method calculates a likelihood ratio test statistic to compare the inside and outside of a scanning window. Areas in the scanning window, that maximized the test statistic, were identified as the most likely clusters. Monte Carlo hypothesis testing is typically used to obtain a p-value for testing the statistical significance of the most likely cluster. Spatial scan statistics have been developed for various probability models such as Poisson [6], Bernoulli [6], normal [7, 8], ordinal [9], and multinomial [10]. The spatial scan statistic method, based on these models, is available through software SaTScan™ [11]. The method has been extended to a regression modeling approach with different regression coefficients for cluster detection [12–14].

Public health surveillance [15] is conducted to collect, analyze, and interpret health-related data for planning, implementing, and evaluating public health policies. As part of the public health surveillance, health-related data were collected from population-based surveys. The data obtained from these ongoing surveys can be used to understand trends in public health [16]. Such health surveys are often based on complex sampling [17] approaches, including several design features such as stratification, cluster sampling, and disproportionate sampling. Sample design features need to be incorporated into the estimation and analysis to generalize the results to the entire population. Therefore, to ensure that the estimation and analysis are generalizable to the entire population, it seems appropriate to consider sample designs and sampling weights when exploring the spatial cluster patterns with the spatial scan statistic.

Some studies have conducted geographic cluster detection analysis using spatial scan statistics on population-based health survey data. However, most of these studies utilized observed survey responses, without considering sample designs and sampling weights. Roberson et al. [18] identified spatial clusters of high stroke prevalence using the spatial scan statistic under the discrete Poisson probability model for a population-based health survey (Behavioral Risk Factor Surveillance System). They specified the number of stroke cases in each county, derived from the observed binary responses, as the case variable in the analysis. Kebede et al. [19] conducted a study to identify spatial clusters of high health coverage among women aged 15‒49 years, using the Bernoulli-based spatial scan statistic on a population-based health survey (Ethiopian Demographic and Health Survey). Similarly, they specified the number of health coverage cases observed for a binary response as the case variable in the analysis.

Two approaches are available for utilizing the survey responses observed with binary outcomes. One approach is to use individual-level data as is, observed with binary responses represented by 0 and 1. In this case, spatial cluster detection can be conducted using the Bernoulli-based spatial scan statistics [6]. The other approach is to use aggregate-level data, which summarizes the individual-level data into regional-level rates for each location. The sampling design and weights can be considered when calculating the region-level rates. For this type of data, spatial cluster detection can be conducted using the weighted normal spatial scan statistic [8], which is used to identify clusters with high rates of regional measures (e.g., mortality rate and disease prevalence at the regional level) with a heterogeneous population.

It is unclear which model is appropriate for use when health survey data comes from a complex survey design. We can use individual-level or aggregated data at the regional level for spatial cluster detection of disease prevalence. The weighted frequency by the sampling weights can be used as binary data to properly consider the sampling design. First, we applied different approaches to the Korea Community Health Survey (KCHS), which is one of the several population-based health surveys in South Korea. We identified statistically significant spatial clusters with high rates of male diabetes diagnoses. Having found that the cluster detection results were very different depending on the type of data, we conducted a simulation study to examine which approach is more appropriate among the three approaches using sampled data from hypothetical population data. Several design features were taken into account when generating the simulation data to mimic real health survey data, such as stratification with different sampling proportions and post-stratification weights. We compared the accuracy of detected clusters in terms of sensitivity and positive predictive value.

## The Korea Community Health Survey (KCHS) data

The KCHS has been conducted annually by the Korea Disease Control and Prevention Agency since 2008 to investigate both public health status and health behaviors at community health centers [20]. KCHS data were collected from an average of 900 adults per community health center (“si/gun/gu” or district level). The survey is based on a complex sample design. Survey data with sample weights can be provided upon request at https://chs.kdca.go.kr/chs.

**Fig. 1:**
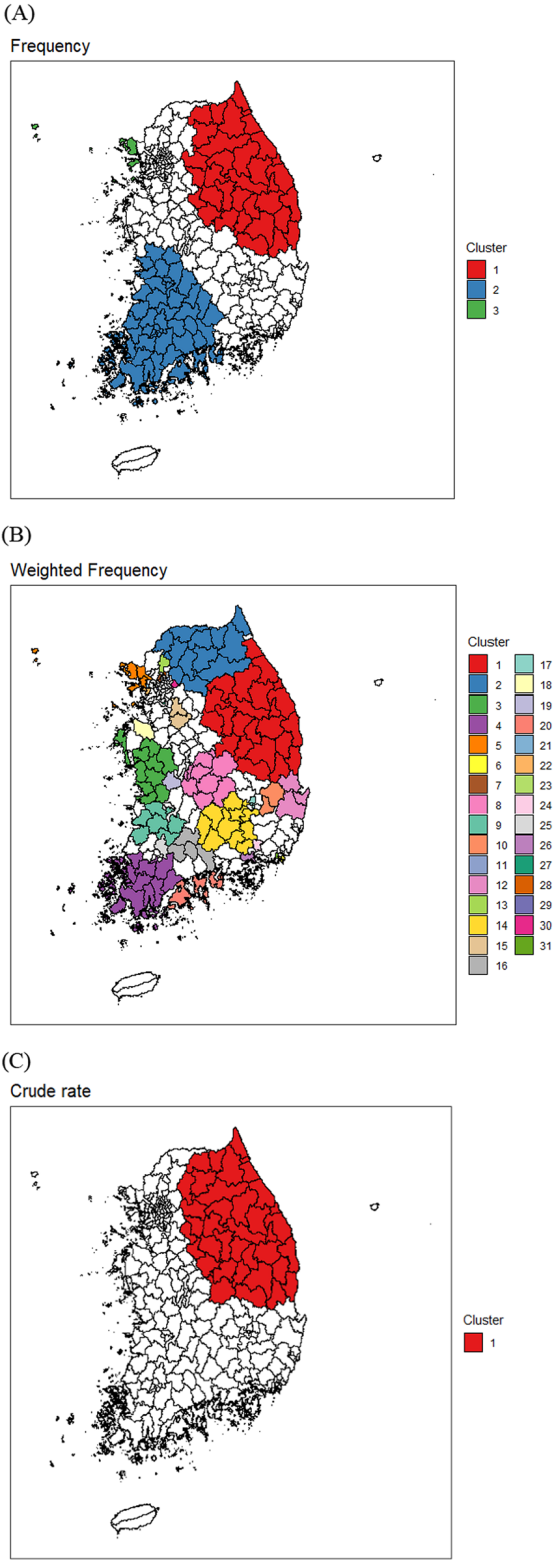
Significant spatial clusters detected with high diabetes diagnosis rates of male adults using the KCHS 2018 data. **A** Individual-level data (frequency). **B** Individual-level data (weighted frequency). **C** Aggregate-level data (crude rate)

**Fig. 2:**
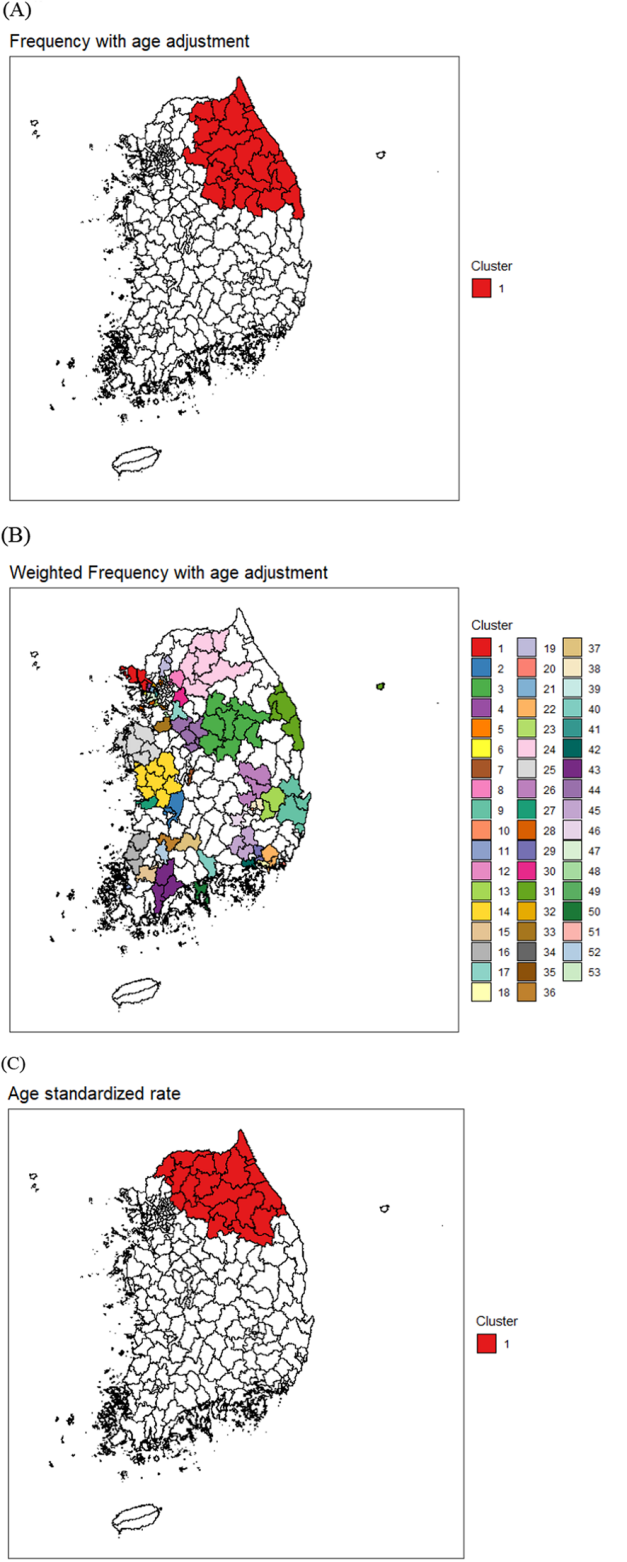
Significant spatial clusters detected with high diabetes diagnosis rates of male adults with age adjustment using the KCHS 2018 data. **A** Individual-level data (frequency with age adjustment). **B** Individual-level data (weighted frequency with age adjustment). **C** Aggregate-level data (age standardized rate)

We used the answers to diagnose diabetes as an outcome from the 2018 KCHS to search for geographic clusters with high rates of diabetes prevalence. There were 250 administrative districts in South Korea in 2018, with the exception of two districts located on Jeju Island. Spatial cluster detection analysis was conducted on the (1) individual-level data, (2) weighted individual-level data, and (3) aggregated data. The Bernoulli-based and weighted normal spatial scan statistics were used for the first two and third data types, respectively. We used the circular scanning window shape and optimal maximum reported cluster size (MRCS) determined by the Gini coefficient [21], while the maximum scanning window size (MSWS) was fixed at 50%. The study participants were divided into male and female subgroups for analysis. All analyses were conducted using the SaTScan™ software version 9.6. This study only shows the results for men. Figures 1 and 2 show cluster detection results for the three different approaches, with and without age adjustment. Only statistically significant clusters were reported at the significance level of 0.05. Tables 1 and 2 include the number of identified high diabetes diagnosis rate spatial clusters at the optimal MRCS value.

**Table 1:** The number of significant clusters detected with high diabetes diagnosis rates of male adults at optimized value of the MRCS when using different types of data from the KCHS 2018 data (see Fig. 1)

Type of data	MRCS	Number of significant clusters
Individual-level data		
Frequency	20	3
Weighted frequency	4	31
Aggregate-level data		
Crude rate	15	1

**Table 2:** The number of significant clusters detected with high diabetes diagnosis rates of male adults with age adjustment at optimized value of the MRCS when using different types of data from the KCHS 2018 data (see Fig. 2)

Type of data	MRCS	Number of significant clusters
Individual-level data		
Frequency	10	1
Weighted frequency	2	53
Aggregate-level data		
Age-standardized rate	10	1

The detected clusters were very different, depending on the approach. These results motivated the present study. With or without age adjustment, the weighted normal model of the aggregated data found a single significant cluster in the northeast area of South Korea. When dealing with the survey data, it is necessary to consider sampling weights for proper inference. One may think that it would be more appropriate to use weighted data by sampling weights rather than observed individual data. However, the Bernoulli model identified too many significant clusters in the weighted data, which could be due to the inflated sample size. Using the survey data, the detected clusters from the Bernoulli model were similar to those based on aggregated data to a certain degree. Only one significant cluster, whose location was similar to that of the cluster detected from the weighted normal model, was detected for the data with age adjustment. Without age adjustment, the most likely cluster was similar to that from the weighted normal model; however, another significant cluster was also detected in the southwest area.

We expected to discover common geographic patterns regardless of the data type used from the survey data. However, the significant spatial clusters with high rates of diabetes diagnosis varied depending on the type of data. The patterns of spatial cluster detection results were similar when using other health outcomes in the 2018 KCHS data. Thus, we aimed to assess which data type derived from binary survey responses is more appropriate for spatial cluster detection using the spatial scan statistic through a simulation study.

## Simulation study

A simulation study was performed to investigate which type of data [individual-level data (frequency and weighted frequency) and aggregate-level data (crude rate estimates)] obtained from the complex sample survey is more appropriate for spatial cluster detection with the spatial scan statistic. First, we generated a hypothetical population dataset based on the administrative districts in South Korea in 2018. The study area consisted of 250 districts. We then sampled 100 iterations from the hypothetical population dataset in a manner similar to the KCHS sampling procedure. Finally, we computed the weighted frequency (individual-level data) and crude rate estimates (aggregate-level data) for each sample dataset using SAS software [22] version 9.4, based on the sample design and sampling weights. For each iteration, we applied the Bernoulli-based spatial scan statistic [6] to two types of individual-level data and the weighted normal spatial scan statistic [8] to aggregate-level data derived from the simulated sample dataset. Age adjustment was not considered in this simulation study. Similar to the KCHS analysis, we only identified statistically significant clusters.

Here, we briefly review the sampling procedure of KCHS, which is based on a complex sample design that uses a two-stage stratified cluster sampling procedure. The surveyed population was stratified by the smallest administrative unit (“dong/eup/myeon”) and housing unit (general house/apartment), which were the first and second strata, respectively. In the first stage, a sample area (“tong/ban/ri”), as a primary sampling unit, was selected for each housing unit type within each administrative unit, based on the number of households through probability proportional to size sampling. In the second stage, households were selected through systematic sampling. The detailed sampling procedure is described in a brief report describing the survey [20].

Sensitivity and positive predictive value (PPV) were used to evaluate the accuracy of the simulation results. Sensitivity was defined as the number of districts included in significant clusters among districts belonging to the true cluster. PPV was defined as the number of districts belonging to the true cluster among the districts included in significant clusters. The average and standard deviation of sensitivity and PPV over 100 iterations are presented. This simulation study was performed using R software [23] version 4.0.2 with the rsatscan package [24] to iteratively run the SaTScan™ software in R environment.

### Population data generation

**Fig. 3:**
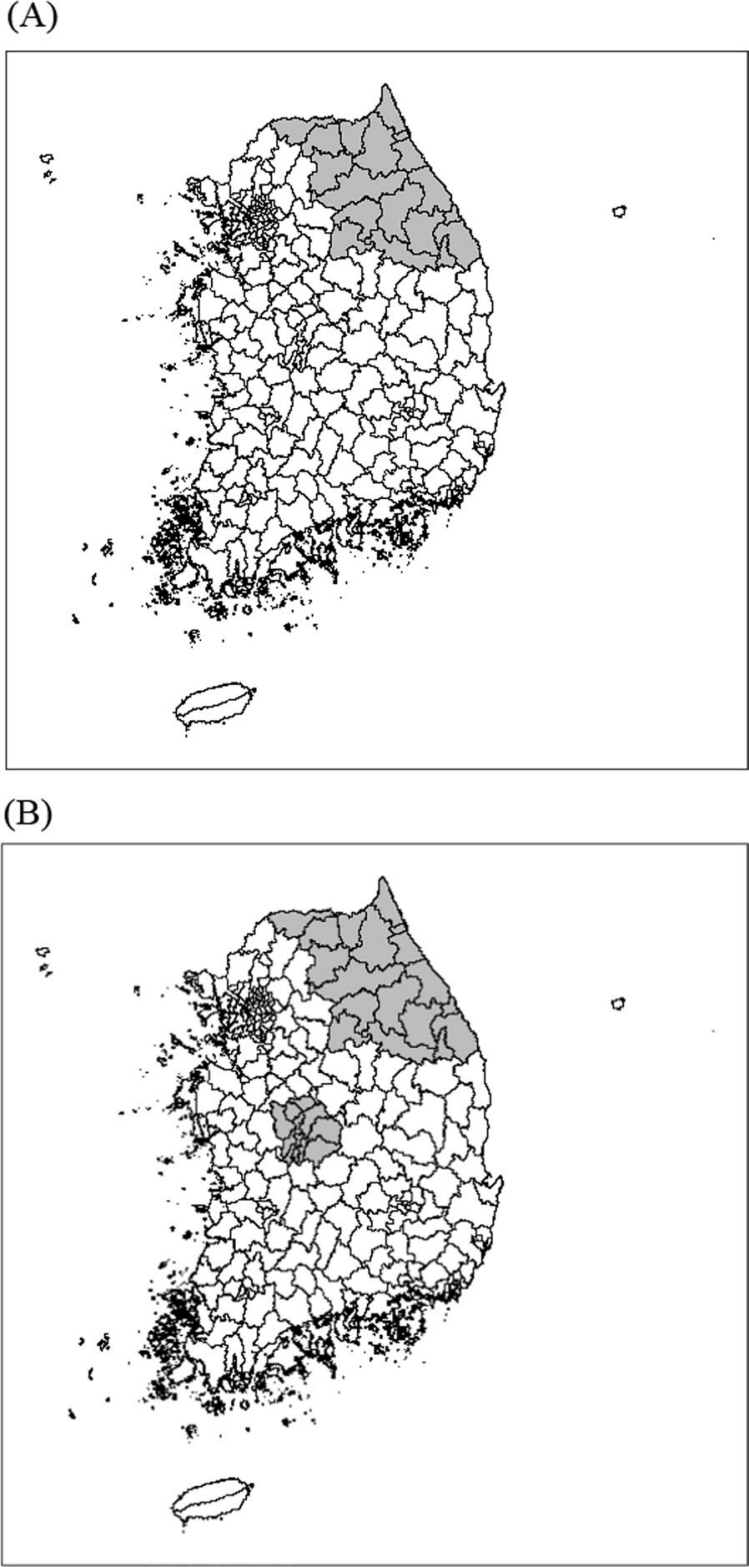
The true simulated cluster models among the 250 districts of South Korea. True cluster model (**A**). True cluster model (**B**)

- (Step1) It was assumed that the population was stratified by age group (20‒34 years, 35‒49 years, 50‒64 years and over 65 years) and sex. Stratification by age group and sex was denoted by $j$ ($j$ = 1 for 20‒34 years of male, 2 for 35‒49 years of male, 3 for 50‒64 years of male, 4 for 65+ years of male, 5 for 20‒34 years of female, 6 for 35‒49 years of female, 7 for 50‒64 years of female, and 8 for 65+ years of female).
- (Step2) We defined two true cluster models with different sizes and shapes using a geographical map of South Korea for 2018. The two true cluster models are shown in Fig. 3. The true cluster in Model (A) was composed of 18 districts located in the northeast, including the coastal areas. We assumed two true clusters in Model (B), one cluster identical to Model (A) and another composed of 12 districts located in the central region. The prevalence rate was set to 0.3 for each district belonging to the true clusters and 0.2 for each district not belonging to the true clusters.
- (Step3) For each district, we generated binary outcomes for individuals from a binomial distribution with the actual population of South Korea in 2018 and the prevalence rate defined in Step2. Binary outcomes were generated from the binomial distribution ${\text{B}}\left( {N_{kj} ,{ }p_{kj} } \right)$, where $N_{kj}$ and $p_{kj}$ denote the actual population and prevalence rate, respectively, for $j{\text{th}}$ stratification of $k{\text{th}}$ district.

### Sample data generation

- (Step1) We defined the sample size for each district ($n_{k}$) between 900 and 920.
- (Step2) The sample size ($n_{kj}$) for each stratification of each district was drawn from a multinomial distribution, with the sample size ($n_{k}$) defined in Step1 and the sampling proportion ($q_{kj}$). The assumed sampling proportions are listed in Table 3. In sampling proportion scenario (1), simple random sampling (SRS) was assumed, which means that $q_{kj}$ was calculated using $N_{kj} /N_{k}$. In sampling proportion scenario (2), we used the actual proportion of the 2018 KCHS by age group and sex as the sampling proportion. In sampling proportion scenario (3), we set a higher sampling proportion of 35‒49 years and 50‒64 years while setting a lower sampling proportion of 20‒34 years and over 65 years for both males and females. This indicates that the sampling proportion in Scenario (3) was more dispersed than actual proportion of the 2018 KCHS [i.e. the sampling proportion scenario (2)]. Through this scenario, we considered a situation where certain groups of the population were more or less likely to be sampled than others, which could cause sampling bias.
- (Step3) We randomly sampled $n_{kj}$ from the hypothetical population dataset for each stratification of each district.
- (Step4) The sampling weight ($w_{kj}$) of a sampled individual for district $k$ and stratification $j$ was calculated as the inverse of the probability that this sampled individual was to be selected. The sampling weight was expressed as follows:
  $$w_{kj} = \frac{{N_{kj} }}{{n_{k} \times q_{kj} }} .$$

**Table 3:** Three different sampling proportions $\left( {\left\{ {q_{kj} } \right\}} \right)$ used in the simulation

Sampling proportion scenarios	Sex	Age groups			
		20‒34 years	35‒49 years	50‒64 years	Over 65 years
(1)	Male	SRS	SRS	SRS	SRS
	Female	SRS	SRS	SRS	SRS
(2)	Male	0.07	0.11	0.14	0.13
	Female	0.08	0.12	0.16	0.19
(3)	Male	0.03	0.15	0.18	0.09
	Female	0.04	0.16	0.20	0.15

*SRS* simple random sampling

The sampling weight ($w_{kj}$) was then adjusted using a post-stratification weight. The post-stratification weight was calculated as the ratio of the actual population from the 2018 Korean census to the sum of the sampling weights by age group and sex for each district. As assumed in the population data generation, we used stratification by age group and sex divided into eight stratifications. The post-stratification weight was calculated as follows:

$$w_{kj}^{post\text{-}stratification} = \frac{{N_{kj} }}{{n_{kj} \times w_{kj} }} = \frac{{N_{kj} }}{{\hat{N}_{kj} }} .$$

Finally, the final sampling weight ($w_{kj}^{final}$) was calculated as follows.

$$w_{kj}^{final} = w_{kj} \times w_{kj}^{post{\text{-}}stratification} .$$

The sampling procedure of the simulation study was conducted according to that described by Vandendijck et al. [25].

### Results of simulation study

The simulation results were obtained for each combination of the true cluster model and sampling proportion scenario (two true cluster models and three sampling proportion scenarios). The average and standard deviation of sensitivity and PPV are presented in Table 4.

**Table 4:** The average sensitivity and PPV (standard deviation in parentheses) over 100 iterations for each data type used for spatial cluster detection under six simulation scenarios

Type of data	Individual-level data		Aggregate-level data
	Frequency	Weighted frequency	Crude rate
Scenario 1 [true cluster (A) + sampling proportion (1)]			
Sensitivity	0.9214 (0.0425)	0.9381 (0.0536)	**0.9435** (0.0055)
PPV	0.8071 (0.1312)	0.1966 (0.0187)	**0.9980** (0.0200)
Scenario 2 [true cluster (A) + sampling proportion (2)]			
Sensitivity	0.9320 (0.0406)	0.9286 (0.0607)	**0.9440** (0.0000)
PPV	0.7861 (0.1255)	0.1889 (0.0163)	**0.9937** (0.0251)
Scenario 3 [true cluster (A) + sampling proportion (3)]			
Sensitivity	0.9203 (0.0379)	0.9280 (0.0549)	**0.9435** (0.0055)
PPV	0.7999 (0.1186)	0.1852 (0.0193)	**0.9994** (0.0059)
Scenario 4 [true cluster (B) + sampling proportion (1)]			
Sensitivity	0.9163 (0.0350)	**0.9378** (0.0402)	0.9192 (0.0218)
PPV	0.8734 (0.0752)	0.3865 (0.0370)	**0.9797** (0.0240)
Scenario 5 [true cluster (B) + sampling proportion (2)]			
Sensitivity	**0.9203** (0.0299)	0.9151 (0.0479)	0.9189 (0.0202)
PPV	0.8749 (0.0766)	0.3640 (0.0419)	**0.9795** (0.0221)
Scenario 6 [true cluster (B) + sampling proportion (3)]			
Sensitivity	0.9183 (0.0339)	0.9244 (0.0476)	**0.9286** (0.0252)
PPV	0.8678 (0.0765)	0.3509 (0.0379)	**0.9708** (0.0255)

The largest values of average sensitivity and PPV for each scenario are in bold

The simulation results showed a similar tendency for the average and standard deviation of sensitivity and PPV in all scenarios. The average sensitivity was generally high in all scenarios, regardless of use of the three types of data, while the average PPV was the highest in all scenarios when using the summary measure (crude rate estimates) at the aggregate-level data. Although the difference was not large, the average sensitivity for the aggregated data was the highest in four of six scenarios. Interestingly, the average PPV was very low when using the weighted frequency compared with the frequency and crude rate estimates. We found that a very large number of clusters were identified throughout the entire study area when using the weighted frequency, as seen in the real data analysis of the KCHS 2018. Also, when using the aggregate-level data, the standard deviation of sensitivity and PPV was relatively low across all scenarios, which implies that we can obtain more consistent and stable results than when using the other approaches. Using the aggregated data from the complex survey seemed to reflect the true spatial cluster patterns better than using other types of data.

## Discussion

In this study, we examined which approach is more appropriate for spatial cluster detection, using data from a population-based health survey. We found that the detected geographic cluster patterns of high disease prevalence varied depending on the type of data, when analyzing the KCHS data. To investigate which data type is more appropriate for spatial cluster detection using spatial scan statistics, we conducted a simulation study. Our findings through the simulation study revealed that the use of area-level summary measure estimates is better at detecting spatial clusters with spatial scan statistics under various scenarios. In all scenarios, although the average sensitivity was similarly high regardless of the use of the three types of data, the average PPV was the highest when using the area-level rate estimates. Therefore, it seems that it is more appropriate to use summary measure estimates (aggregate-level data), which takes the sample design and sampling weights into account, for geographical cluster detection with the spatial scan statistic than the other types of data.

One limitation of this study is that we partially implemented the sampling procedure of KCHS in the simulation study. KCHS is based on a two-stage stratified cluster sampling procedure; however, we could not consider the cluster sampling features to simplify the simulation process. Nevertheless, this simplified sampling procedure appears to yield meaningful results because the sampling weights are available in the sample data sampled from the hypothetical population data.

## Conclusion

Based on our findings from the simulation study, it seems that it is more appropriate to use aggregate-level data (the rate estimates) among the three types of data from the population-based health survey, when exploring spatial cluster detection with the spatial scan statistic. It is expected that more simulation studies will need to be performed by considering other sampling features, such as cluster sampling, to obtain more comprehensive results.

## Data Availability

The datasets used and/or analyzed during the current study are available from the corresponding author on reasonable request.

## Abbreviations

MRCS: Maximum reported cluster size
MSWS: Maximum scanning window size
KCHS: Korea Community Health Survey
PPV: Positive predictive value
SRS: Simple random sampling
